# Magnetic hysteresis in self-assembled monolayers of Dy-fullerene single molecule magnets on gold†
†Electronic supplementary information (ESI) available. See DOI: 10.1039/c8nr00511g


**DOI:** 10.1039/c8nr00511g

**Published:** 2018-05-23

**Authors:** C.-H. Chen, D. S. Krylov, S. M. Avdoshenko, F. Liu, L. Spree, R. Westerström, C. Bulbucan, M. Studniarek, J. Dreiser, A. U. B. Wolter, B. Büchner, A. A. Popov

**Affiliations:** a Leibniz Institute for Solid State and Materials Research (IFW) , D-01069 Dresden , Germany . Email: a.popov@ifw-dresden.de; b The Division of Synchrotron Radiation Research , Lund University , SE-22100 Lund , Sweden; c Swiss Light Source , Paul Scherrer Institute , CH-5232 Villigen PSI , Switzerland

## Abstract

Fullerene single molecule magnets (SMMs) DySc_2_N@C_80_ and Dy_2_ScN@C_80_ are functionalized *via* a 1,3-dipolar cycloaddition with surface-anchoring thioether groups. The SMM properties of Dy-fullerenes are substantially affected by the cycloaddition. Submonolayers of the physisorbed derivatives exhibit magnetic hysteresis on an Au(111) surface at 2 K as revealed by X-ray magnetic circular dichroism.

## Abstract 2

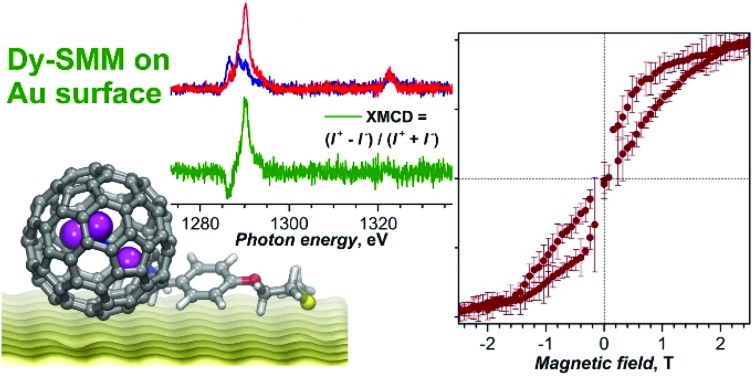
Self-assembled monolayers from single molecule magnets DySc_2_N@C_80_ and Dy_2_ScN@C_80_ functionalized with thioether groups retain magnetic bistability on Au(111) surface.

## 

Molecules with a bistable magnetic ground state and a slow relaxation of magnetization are known as single molecule magnets (SMMs).^1^ Information storage or spintronic applications envisaged for SMMs require contacting the molecules to electrodes, and observing whether hysteresis with remanence is retained on metal is important for the electrically readable magnetic bits. The magnetic properties of SMMs on conducting surfaces should therefore be well understood. However, this problem meets with serious difficulties in both bringing the molecules to metallic substrates and in studying the magnetic properties of monolayers.^2^ As a result, the number of molecules that have been studied on different surfaces is very low compared to the number of published SMMs, and hysteresis of magnetization in monolayers deposited on metals has been observed so far only for TbPc_2_,^3^ Fe_4_,^4^ and Dy_2_ScN@C_80_.^5^

Evaporation is the most popular way to obtain monolayers of SMMs. Unfortunately, only a limited number of SMMs are sublimable and retain their structural integrity on a surface.4c,^5^,^6^ Chemical deposition from solution *via* covalent bonding to the substrate, such as realized in a self-assembled monolayer (SAM) approach, is a viable alternative to vapour deposition. It avoids the thermal stability problem and allows the formation of monolayers in a comparably simple self-limiting procedure. Functionalization of SMM molecules with surface-anchoring groups and formation of SAMs on metals has previously been studied for Mn_12_,^7^ TbPc_2_,^8^ Fe_4_,4a,b,^9^ and Fe_3_Cr.^10^ SAMs of Fe_4_ were the first SMMs to exhibit magnetic bistability on a metallic substrate.4a,b

In this work, we explore the SAM approach for the deposition of magnetic fullerenes onto an Au(111) surface and study the magnetic properties of the derivatives and SAMs. Dy-based endohedral metallofullerenes (EMFs) have been found to be robust SMMs, with high anisotropy barriers or giant exchange interactions, and reasonably high blocking temperature of magnetization in bulk materials.^11^ The reports on EMF-SAMs are limited so far to non-SMM Er_3_N@C_80_ and La@C_82_,^12^ but SAMs of empty fullerenes are relatively well known and have been studied in great detail.^13^ An STM study of a series of C_60_-based SAMs on Au(111) with thiol and thioether anchoring groups revealed that the latter provided physisorbed monolayers with more ordered packing of molecules than in chemisorbed thiol derivatives.^14^

DySc_2_N@C_80_-*I*_*h*_ (**1**) and Dy_2_ScN@C_80_-*I*_*h*_ (**2**) were obtained as described before.11f,^15^ EMFs were functionalized with a thioether –S–CH_3_ group *via* 1,3-dipolar cycloaddition (Fig. 1) adopting a procedure developed for C_60_-SAMs.^14^ The optimal reaction time of 20 min was first determined for Sc_3_N@C_80_ (longer times resulted in the formation of bis-adducts) and then used for **1**. **2** exhibited lower reactivity and was reacted for 40 minutes. Fig. 1b shows HPLC traces of the reaction mixtures, as well as HPLC traces of isolated derivatives (denoted **1-R** and **2-R** hereafter). A partial formation of simple fulleropyrrolidino adducts was also observed (denoted by asterisks in Fig. 1b).

**Fig. 1:**
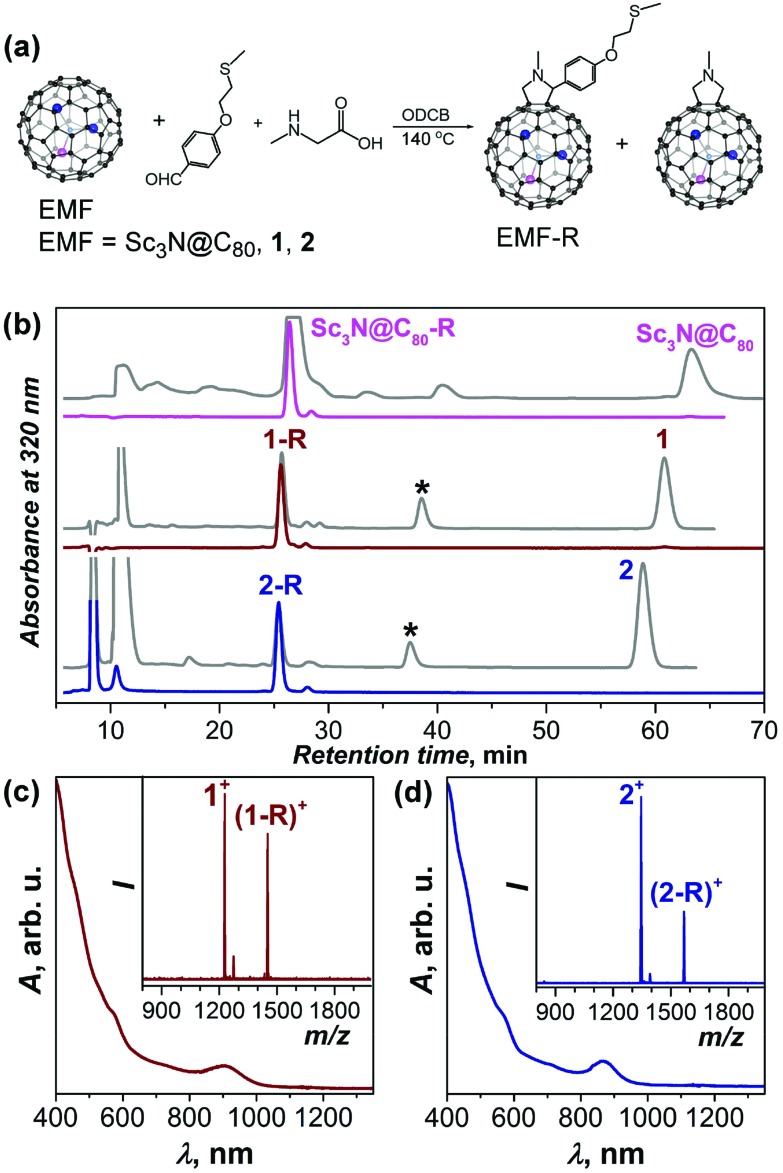
(a) Scheme of a Prato reaction to obtain EMF-R derivatives (EMF = Sc_3_N@C_80_, DySc_2_N@C_80_ (**1**), and Dy_2_ScN@C_80_ (**2**)); (b) HPLC curves measured at the end of the reaction (grey traces) and for purified derivatives (coloured curves). The absence of the pristine fullerenes (HPLC peaks near 60 min) in the purified derivatives can be clearly seen; (c) Vis-NIR absorption spectra of **1-R** and **2-R** in toluene. Insets show MALDI mass-spectra (1,1,4,4-tetraphenyl-1,3-butadiene as a matrix). Despite considerable fragmentation to pristine fullerenes, molecular ions of **1-R** and **2-R** can be clearly seen.

Prato addition to M_3_N@C_80_-*I*_*h*_ nitride clusterfullerenes with Sc_3_N and mixed-metal clusters gives predominantly [5,6]-fulleropyrrolidino cycloadducts (*i.e* the addition proceeds across a pentagon/hexagon edge).^16^ The structural identity of isolated **1-R** and **2-R** as [5,6]-monoadducts was established by MALDI and vis-NIR absorption spectroscopy (Fig. 1c and d). For Sc_3_N@C_80_-R, the molecular structure was additionally confirmed by 1D and 2D ^1^H NMR spectroscopy (Fig. S1–S3†). Paramagnetic temperature-dependent ^1^H NMR spectra were also measured for **1-R** (Fig. S4†). Vis-NIR absorption spectroscopy of the derivatives showed the characteristic pattern of [5,6]-adducts; the spectra of all three derivatives are similar except for the lowest-energy transition, the energy of which increases with the number of Dy atoms from 1.29 eV (960 nm) in Sc_3_N@C_80_-R to 1.38 eV (900 nm) in **1-R** and 1.43 eV (865 nm) in **2-R** (see also Fig. S5†).

The magnetic properties of powder samples of **1-R** and **2-R** were first studied by SQUID magnetometry and compared to pristine **1** and **2**. Both derivatives showed magnetic hysteresis at low temperatures (Fig. 2). **1-R** exhibits the butterfly-shape hysteresis with an abrupt drop of magnetization in zero field, which is characteristic for quantum tunneling of magnetization and is often observed in single-ion Dy-SMMs, including **1**.11d,e,^17^ At the field sweep rate of 2.9 mT s^–1^, hysteresis in **1-R** closes above 8 K, which is somewhat higher than in the pristine **1**. Similarly, the blocking temperature of magnetization (*T*_B_), determined as a position of the peak in the magnetic susceptibility of a zero-field cooled sample, is 8 K for **1-R** and 7 K for **1** 11*g* (Fig. 2b). The relaxation times of **1** and **1-R** measured in a finite field of 0.2 T between 1.8 and 5 K are similar and vary from *ca.* 100 s near 5 K to 10^4^ s near 2 K (Tables S1 and S2†). The temperature trend of relaxation times is slightly different for **1** and **1-R**, the latter showing a steeper temperature decay (Fig. S6†). To summarize, based on the magnetization behaviour and relaxation times, we conclude that cycloaddition improves low-temperature SMM properties of **1** (a similar effect was also reported in ref. 18).

**Fig. 2:**
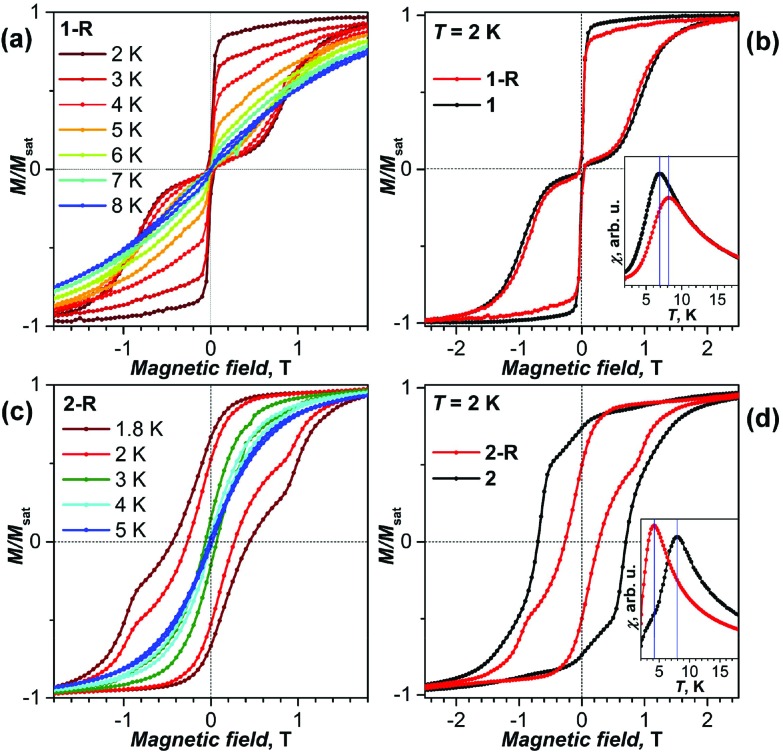
(a) Magnetization curves of **1-R** at temperatures 2–8 K, sweep rate 2.9 mT s^–1^. (b) Magnetization curves of **1-R** and **1** at *T* = 2 K; the inset shows determination of the blocking temperatures of magnetization *T*_B_, sweep rate 5 K min^–1^. (c) Magnetization curves of **2-R** at temperatures 1.8–5 K, sweep rate 2.9 mT s^–1^; (d) magnetization curves of **2-R** and **2** at *T* = 2 K. The inset shows determination of *T*_B_, sweep rate 5 K min^–1^.

The influence of cycloaddition on **2** is opposite to that of **1**. **2-R** shows a considerably lower *T*_B_ of 4 K (*vs*. 8 K in **2** 11*f*), its hysteresis is narrower with the coercive field of 0.27 T at 2.0 K (*vs*. 0.70 T in **2**) and is essentially closed above 5 K. Zero-field relaxation times of magnetization of 184 s and 55 s were determined for **2-R** at 1.8 and 2.0 K, respectively (at higher temperatures the times are too short for reliable measurement by dc-SQUID magnetometry). These values allow estimation of the universal SMM parameter *T*_B100_, the temperature at which the relaxation time is 100 s, at 1.9 K. In **2**, relaxation times at 1.8 and 2.0 K are 5100 and 2360 s, respectively, and *T*_B100_ is near 5 K.11*f* Thus, derivatization substantially worsens the low-temperature SMM properties of **2**.

The magnetization behaviour of **1-R** and **2-R** shows that although the magnetic cluster is hidden inside the carbon cage, exohedral derivatization of the fullerene induces pronounced effects on the low-temperature SMM properties. Below 10 K, relaxation of magnetization in EMF-SMMs does not proceed *via* the Orbach mechanism involving crystal-field excited states of Dy ions, but rather involves low-energy exchange excited states and localized phonons strongly coupled to a spin system.11a,b,f,g Our results indicate that the cluster–cage interactions (which are altered by cycloaddition) have significant influence on the relaxation of magnetization in this low-temperature under-barrier regime. Cycloaddition changes the potential energy surface of the endohedral cluster and hence alters the frequencies of vibrations, corresponding to frustrated rotations and translations of the cluster. It is reasonable to suggest that such vibrations play an important role in the relaxation of magnetization.^19^ Understanding this factor will enable the design of better synthetic strategies for SMMs based on EMF derivatives.

For the preparation of SAMs, Au(111) single crystals were immersed into toluene solutions of **1-R** or **2-R** for 24 hours, and then washed with toluene to remove excess molecules. The samples were then immediately inserted into the fast entry lock of the beam line's vacuum system and subsequently studied by X-ray absorption spectroscopy (XAS), X-ray natural linear dichroism (XNLD), and X-ray magnetic circular dichroism (XMCD) at the Dy-*M*_4,5_ absorption edges as shown in Fig. 3(a and b) and Fig. S8–S11.† Both samples exhibit the multiplet structure typical for the Dy^3+^ state. The XAS signal is rather weak pointing to a low submonolayer coverage, which is determined to be 5–10% of a densely packed layer of unfunctionalized fullerene (Fig. S8†). XNLD measurements of **2-R** did not show any preferential orientation of Dy–N units at room temperature (Fig. S10†). At 2 K and in the magnetic field of 6.5 T, magnetic circular dichroism can be clearly observed in both samples (Fig. 3; the magnetic field is parallel to X-rays). XMCD measurements with normal and grazing incidence of incoming X-rays gave essentially identical dichroism. In nitride clusterfullerenes such as **1** and **2**, Dy ions have very large magnetic anisotropy due to the strong ligand field, mainly imposed by the nitride ion. As a result, the magnetic moments of Dy ions are aligned along the Dy–N bonds. The lack of the angular dependence of XMCD agrees with the negligible XNLD signal and points to the random, disordered orientations of Dy magnetic moments. In other words, it indicates that **1-R** and **2-R** molecules are not ordered on the Au(111) substrate, as opposed to the ordering found in the sub-monolayer of **2** on Rh(111).^5^

**Fig. 3:**
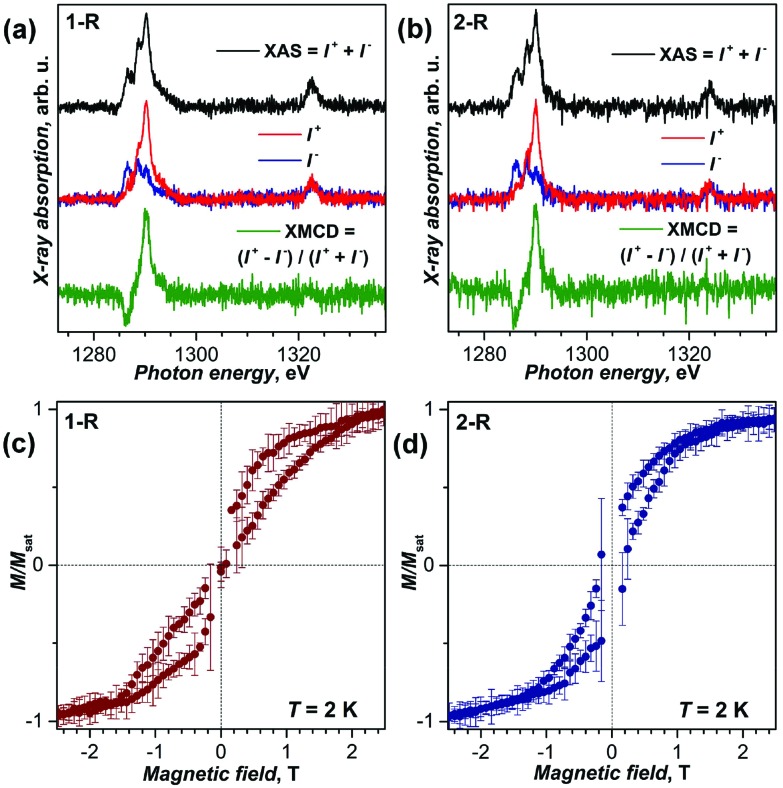
(a, b) X-ray absorption spectra of **1-R** (a) and **2-R** (b) at the Dy-*M*_4,5_ absorption edge measured at 2 K in the magnetic field of 6.5 T; *I*^+^ and *I*^–^ denote right-hand and left-hand circular polarization of incoming X-rays. (c, d) Magnetization curves of sub-monolayers of **1-R** (c) and **2-R** (d) measured by XMCD at 2 K with a sweep rate of 2 T min^–1^ (averaging over five measured curves, error bars are standard deviations).

The XMCD signal at the strongest feature of the *M*_5_ edge of Dy was used to measure the magnetization of the samples at varying magnetic field. Fig. 3c and d show magnetization curves measured for SAMs of **1-R** and **2-R** at 2 K. In both samples, distinct hysteresis of magnetization is observed. Thus, magnetic bistability and slow relaxation of magnetization are observed in SAMs of **1-R** and **2-R** on Au(111). The hysteresis of SAMs is narrower than in the bulk samples (see Fig. S11† for comparison), although the latter were studied with a considerably slower sweep rate. Faster relaxation of magnetization in SAMs is presumably caused by direct contacts of fullerenes with the metallic substrate (see below), but may be also affected by X-ray induced demagnetization.^20^

To clarify the behaviour of **1-R** and **2-R** molecules on the gold surface, DFT calculations and DFT-based molecular dynamics (MD) simulations were performed for an Sc_3_N@C_80_-R′ molecule with a simplified structure of the linker R^21^ positioned on 3 atomic layers of gold. After the start of MD modelling^22^ at 300 K from the vertical position (such as shown in Fig. 4a, see also Fig. S12†), in 2 ps the molecule adopted a configuration with horizontal alignment of the linker and vertical position of the fullerene (Fig. 4b), and after a further 5 ps this structure changed into the fully horizontal configuration (Fig. 4c) with both the fullerene and the linker touching the metal. At 300 K the latter configuration is found to be highly mobile. Over the next 20 ps the molecule exhibited both lateral motions on the surface and rotations of the fullerene core (Fig. S13†), resulting in multiple orientations of the endohedral cluster.^21^

**Fig. 4:**
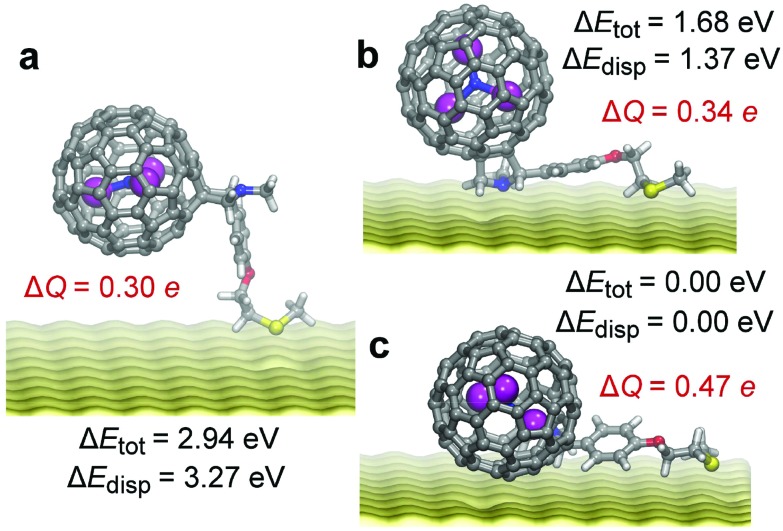
Representative DFT-optimized configuration of Sc_3_N@C_80_-R on gold: (a) vertical configuration; (b) the structure with horizontal alignment of the linker and vertical orientation of the fullerene cage; (c) the structure with fully horizontal alignment of the linker and the fullerene core. Also shown for each structure are relative energies (Δ*E*_tot_), dispersion contribution (Δ*E*_disp_), and the total charge transfer between the Sc_3_N@C_80_-R molecule and the substrate (Δ*Q*).

Selected conformations along the MD trajectory were chosen for further structure optimization at the PBE-D level using the projector augmented-wave method and with the experimental structure of the linker R.^23^ The structures with fully horizontal alignment of the molecule parallel to the surface have the lowest energies, which span the range of *ca.* 0.0–0.3 eV (Fig. 4c shows the lowest energy configuration). The presence of many energy minima with similar energies indicates that freezing of the SAMs with a low degree of coverage will result in multiple orientations of fullerene molecules on the surface. The structures with a horizontal organic tail but vertical fullerene orientation are less stable by 1.7–1.8 eV, whereas the structures with vertical orientation of the molecule are the least stable with relative energies of 2.8–2.9 eV. The main contribution to such a large variation of relative energies is caused by dispersion interactions (Table S3 and Fig. S14†). Note that the conclusion about the preference of the horizontal configuration was also reached for C_60_-based SAMs with analogous thioether linkers.^14^

Analysis of the Bader atomic charges^24^ revealed that the Sc_3_N@C_80_-R molecule transfers 0.3–0.5 electrons to the metallic substrate (the values are referred *versus* the charges of the isolated molecule). The exact number depends on the configuration of the molecule. When Sc_3_N@C_80_ is in direct contact with the substrate, the fullerene-to-substrate charge transfer is the largest, reaching 0.30*e* in the structure shown in Fig. 4c. In the absence of direct contact (Fig. 4a and b), the charge transfer from the Sc_3_N@C_80_ core is reduced to *ca.* 0.15*e*. The linker also transfers *ca.* 0.15*e* to the metal (Table S3 and Fig. S14†). However, irrespective of the configuration of the molecule on the substrate and the magnitude of the total charge transfer, the charge of the endohedral Sc_3_N cluster remains the same (within 0.01*e*) as in the isolated Sc_3_N@C_80_-R molecule. This shows that the π-system of the carbon cage effectively screens the Sc_3_N cluster from the substrate and keeps its charge state intact. The “Faraday cage” effect of the fullerene^21^,^25^ thus helps to preserve the magnetic state of the endohedral cluster even when the outer molecule experiences rather strong interactions with the metallic substrate.

## Conclusions

1,3-Dipolar cycloaddition substantially affects single molecule magnetism of nitride clusterfullerenes, but does so differently for DySc_2_N@C_80_ and Dy_2_ScN@C_80_. The low-temperature SMM properties of DySc_2_N@C_80_ are improved in the cycloadduct, whereas those of Dy_2_ScN@C_80_ are worsened as concluded from the changes in the blocking temperature of magnetization and relaxation times. Surface deposition onto Au(111) from toluene solution gives low-coverage SAMs of both fullerenes with thioether anchoring groups. At 2 K both SAMs exhibit hysteresis of magnetization on gold. However, the strong interaction of the fullerene and the linker with the substrate results in the preference of horizontal configurations, featuring direct contact of the fullerene core with the metallic surface. The structures are mobile at room temperature and freeze in multiple orientations at low temperatures, leading to random orientation of the magnetic moments of endohedral Dy ions.

## Conflicts of interest

There are no conflicts to declare.

## Supplementary Material

Supplementary information
