# Supplementary material for: Magnetic hysteresis in self-assembled monolayers of Dy-fullerene single molecule magnets on gold
Source: Nanoscale. 2018 May 23;10(24):11287–92. doi: 10.1039/c8nr00511g (PMC6018719; doi:10.1039/c8nr00511g)
Supplement: Supplementary file 1 [file NR-010-C8NR00511G-s001.pdf]

Electronic Supplementary Material (ESI) for Nanoscale.
This journal is © The Royal Society of Chemistry 2018

Magnetic hysteresis in self-assembled monolayers of Dy-fullerene single molecule magnets on gold

C.-H. Chen,[a] D. S. Krylov,[a] S. M. Avdoshenko,[a] F. Liu,[a] L. Spree,[a] R. Westerström,[b] C. Bulbucan,[b] M. Studniarek,[c] J. Dreiser,[c] A. U. B. Wolter,[a] B. Büchner,[a] and A. A. Popov*[a]

Supporting Information

**General methods and synthesis of EMF derivatives**	**S2**
**Spectroscopic characterization**	**S4**
**Magnetic properties**	**S7**
**XPS spectra**	**S9**
**Additional XAS data**	**S10**
**Molecular dynamics modelling**	**S14**
**Results of DFT-optimization on Au**	**S15**
**References**	**S17**

General methods

All manipulations were carried out under nitrogen atmosphere. ^{1}H spectra were obtained on a Bruker Avance spectrometer at 500 MHz. UV-Vis-NIR spectra were recorded from 200 to 2000 nm in toluene by using a 1.0 cm quartz cell with a Shimadzu 3100 spectrophotometer. Matrix-assisted laser desorption ionization (MALDI) mass spectra were recorded on a Bruker autoflex mass spectrometer.

SAM growth: Before the use for the SAM growth, Au substrates were sonicated in toluene for 1 hour. Then, the crystals were immersed into toluene solution of the derivatized fullerene (concentration ca 50 µmol/L) for 24 hours. After that, the substrate was taken out, washed with excess of toluene and dried under nitrogen stream. Operation were performed in the laminar flow box.

Synthesis of $Sc_3N@C_{80}$-R

$Sc_3N@C_{80}$ (1.1 mg, 0.001 mmol), N-methyl glycine (1.3 mg, 0.015 mmol) and 4-(2-methylsulfanyl-ethoxy)benzaldehyde (9.7 mg, 0.05 mmol) were placed in an oven-dried 25 mL three-neck round-bottom flask, under a dinitrogen atmosphere. *o*-dichlorobenzene (5 mL) was introduced into the flask via a syringe, and the solution was heated to 140°C for 20 min. The solution was cooled to room temperature, dried under vacuum, and the residue was purified by semi-preparative HPLC with a Buckyprep column (10 × 250 mm), eluting with toluene (flow rate: 1.5 mL min^{-1}). The brown compound $Sc_3N@C_{80}$-R was collected at the retention time of 27 min.

^{1}H NMR {500 MHz, $CDCl_3$, 258 K} δ_H 7.91 (d, 1H, aromatic H), 7.21 (d, 1H, aromatic H), 7.19 (d, 1H, aromatic H), 6.86 (d, 1H, aromatic H), 4.33 (d, 1H, -CH_2 pyrrolidine), 4.26 (tq, 2H, -OCH_2), 3.63 (s, 1H, -CH pyrrolidine), 2.98 (d, 1H, -CH_2 pyrrolidine), 2.97 (S, 2H, -SCH_2), 2.50 (s, 3H, -NCH_3), 2.27 (s, 3H,-SCH_3) ppm. MALDI m/z 1331.981.

Synthesis of $DySc_2N@C_{80}$-R (1-R)

$DySc_2N@C_{80}$ (1 mg, 0.8 x 10^{-3} mmol), N-methyl glycine (1 mg, 0.011 mmol), 4-(2-methylsulfanyl-ethoxy)benzaldehyde (8 mg, 0.041 mmol) and o-dichlorobenzene (5 mL) were introduced in an oven-dried 25 mL three-neck round-bottom flask, under a dinitrogen atmosphere. The solution was heated to 140°C for 20 min. The solution was cooled to room temperature, dried under vacuum, and the residue was purified by semi-preparative HPLC with a Buckyprep column (10 × 250 mm), eluting with toluene (flow rate: 1.5 mL min^{-1}). The brown compound **1-R** was collected at the retention time of 25 min. MALDI m/z 1450.952. HPLC retention time of **1-R** is considerably shorter than the retention of the pristine fullerene **1** (61 min in the same conditions, see Fig. 1b), which ensures that the pristine fullerene is not present in the collected fraction and the ion of $\mathbf{1}^+$ observed in the MALDI mass-spectra (Fig. 1c) is due to the fragmentation of the derivative during the laser evaporation and ionization.

Synthesis of $Dy_2ScN@C_{80}$-R (2-R)

An oven-dried 25 mL three-neck round-bottom flask was charged with $Dy_2ScN@C_{80}$ (1.1 mg, 0.8 x 10^{-3} mmol), N-methyl glycine (1.1 mg, 0.012 mmol) and 4-(2-methylsulfanyl-ethoxy)benzaldehyde (8 mg, 0.041 mmol) under a dinitrogen atmosphere. *o*-dichlorobenzene (5 mL) was introduced into the flask via a syringe, and the solution was heated to 140°C for 40 min. The solution was cooled to room temperature, dried under vacuum, and the residue was purified by semi-preparative HPLC with a Buckyprep column (10 × 250 mm), eluting with toluene (flow rate: 1.5 mL min^{-1}). The brown compound **2-R** was collected at the retention time of 25 min. MALDI m/z 1567.902. HPLC retention time of **2-R** is considerably shorter than the retention of the pristine fullerene **2** (58 min in the same conditions, see Fig. 1b), which ensures that the pristine fullerene is not present in the collected fraction and the ion of $\mathbf{2}^+$ observed in the MALDI mass-spectra (Fig. 1c) is due to the fragmentation of the derivative during the laser evaporation and ionization.

Estimation of the yield

The reactions were monitored by HPLC, which also allowed estimation of the yield of the derivatives **1-R** and **2-R**. Pristine fullerene have much longer retention times (more than 60 minutes in comparison to 25 minutes for the derivatives), which ensures that their peaks do not overlap, and hence separation of **1-R** from unreacted 1 as well as **2-R** from unreacted **2-R** is straightforward. Figure 1b also compares HPLC curves of the reaction mixtures (at the moment when reaction was stopped) and of the derivatives after HPLC separation. The peaks of pristine fullerenes are well seen in the reaction mixtures, but are absent in the separated products. Thus, chromatographic analysis ensures that *pristine fullerene were not present in the samples* of derivatives **1-R** and **2-R**. Based on the HPLC peak areas, we can also estimate the reaction yield of the derivatives. For **1-R** the yield is 27% based on the initial amounts of the fullerene **1**, and 56% based on the conversed fullerene. For **2-R**, the yield is 20% based on the initial amounts of fullerene **2**, and 52% based on the conversed fullerene. The main undesired product (ca 30% yield based on the conversed fullerene) in both cases is the fulleropyrrolidine derivative without the functional group (its HPLC peak is indicated by an asterisk in Fig. 1b). Conversion of the pristine fullerene may be increased by the longer reaction times, but since this also leads to the increase of the yield of bis- and polyadducts and hence substantially complicate the separation, we preferred to stop reaction after 20 minutes for **1** and 40 minutes for **2** as indicated in the manuscript.

^{1}H NMR spectroscopy of EMF-R

The ^{1}H NMR spectrum of **$Sc_3N@C_{80}$-R** at 25 °C (Fig. S1) shows broad signals in the region of 8.2 ~ 6.8 ppm which belong to aromatic protons, and may due to a dynamic effect produced by the restricted rotation of the bulky group.[10] Lowering the temperature to -15 °C, the four doublets are observed at δ = 7.91, 7.21, 7.19 and 6.86 ppm that can be assigned to the four aromatic protons of different chemical environments. The upfield region of the ^{1}H NMR spectrum displays a singlet at δ 3.63 for the methynyl proton, two doublets at δ 4.33 and 2.98 for the diastereotopic methylene protons, and the four signals (δ = 4.26, 2,97, 2.50 and 2.27 ppm) are assigned to the protons in the i, j, d and k positions, respectively (see Fig S2). These assignments are confirmed by the COSY and NOESY spectrum (Fig S3).

^{1}H NMR spectra of **1-R** are shown in Fig. S4. The compound is paramagnetic and exhibits strongly shifted and broadened peaks

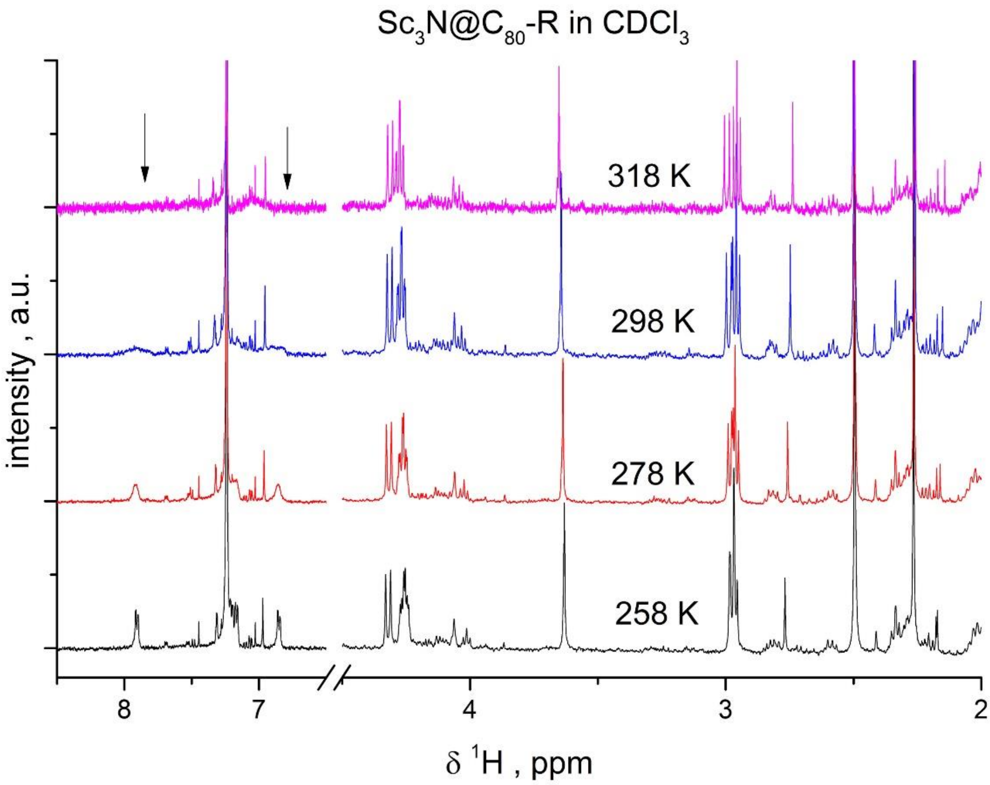

Figure S1. Variable-temperature ^{1}H NMR spectra of $Sc_3N@C_{80}$-R in $CDCl_3$.

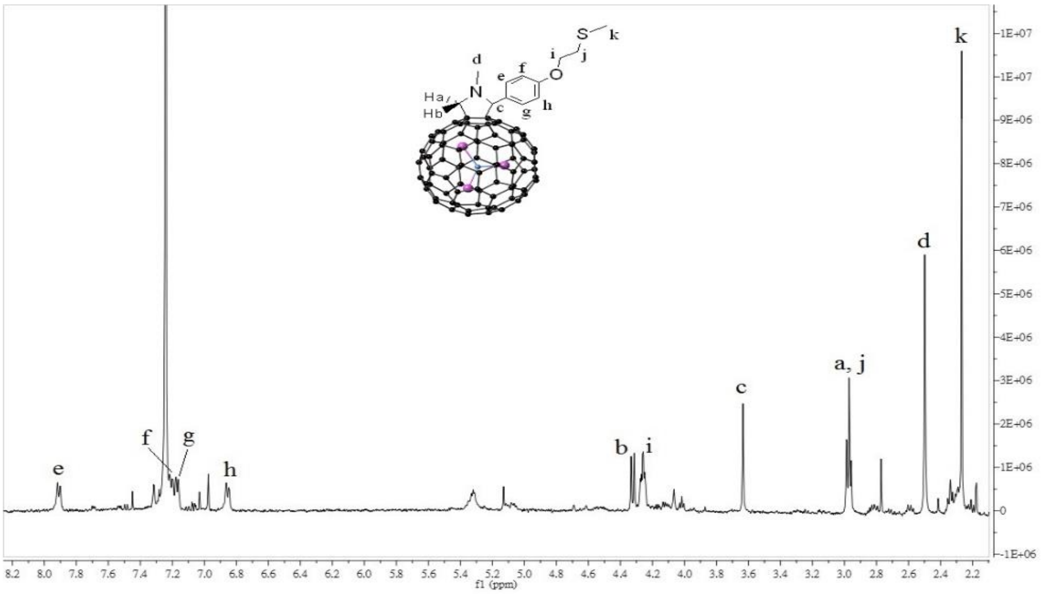

Figure S2. ^{1}H NMR spectrum of $Sc_3N@C_{80}$-R (500 MHz, $CDCl_3$, 258 K) with assignment of the peaks.

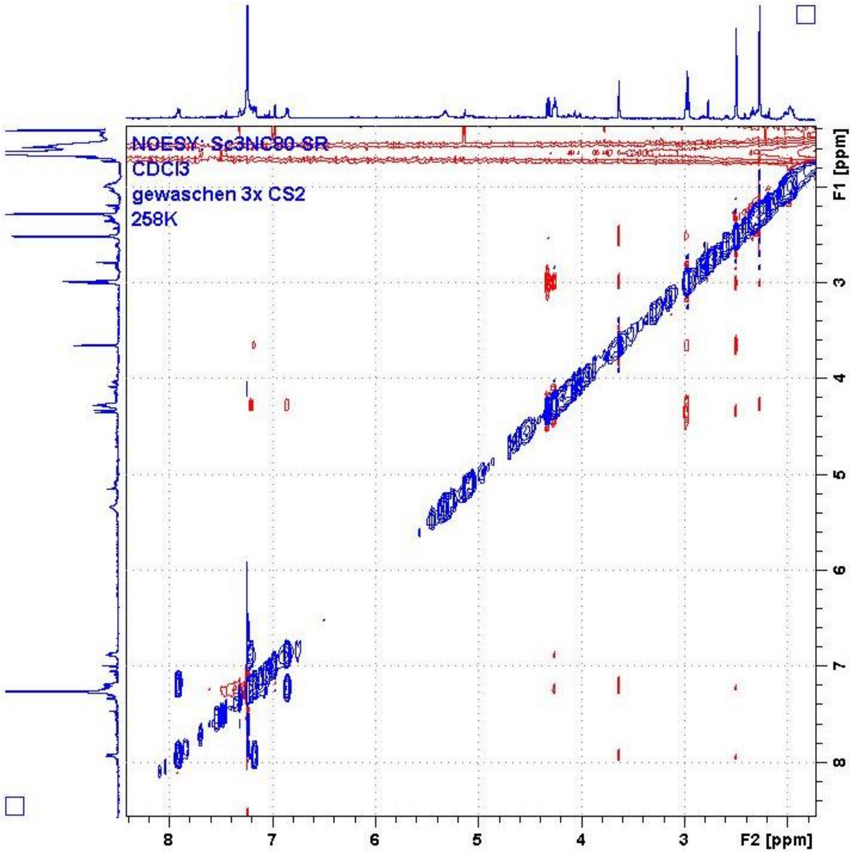

Figure S3. ^{1}H-^{1}H-COSY NMR of mixture of $Sc_3N@C_{80}$-R (blue line); ^{1}H NOESY NMR spectra of $Sc_3N@C_{80}$-R (red line).

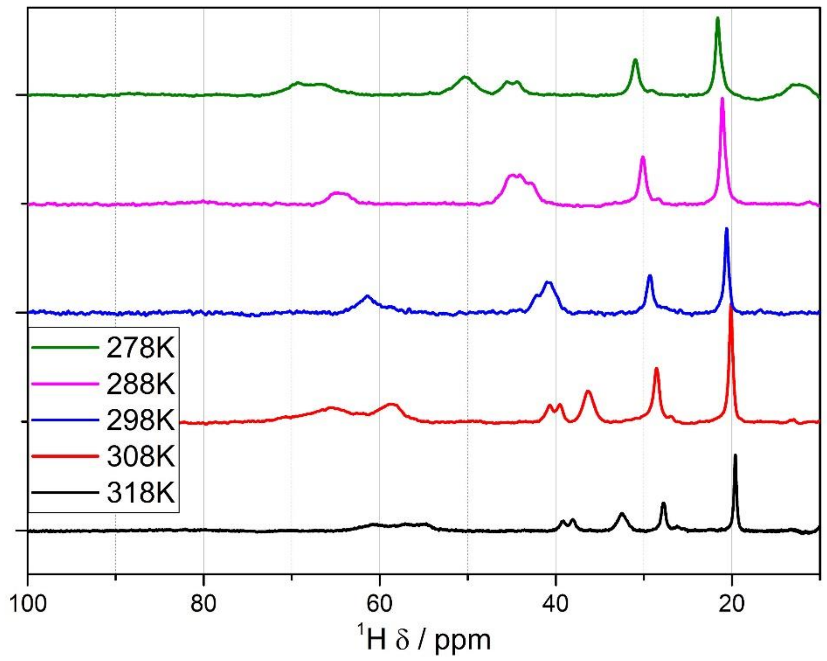

Figure S4. ^{1}H NMR spectra of **1-R** measured at different temperatures.

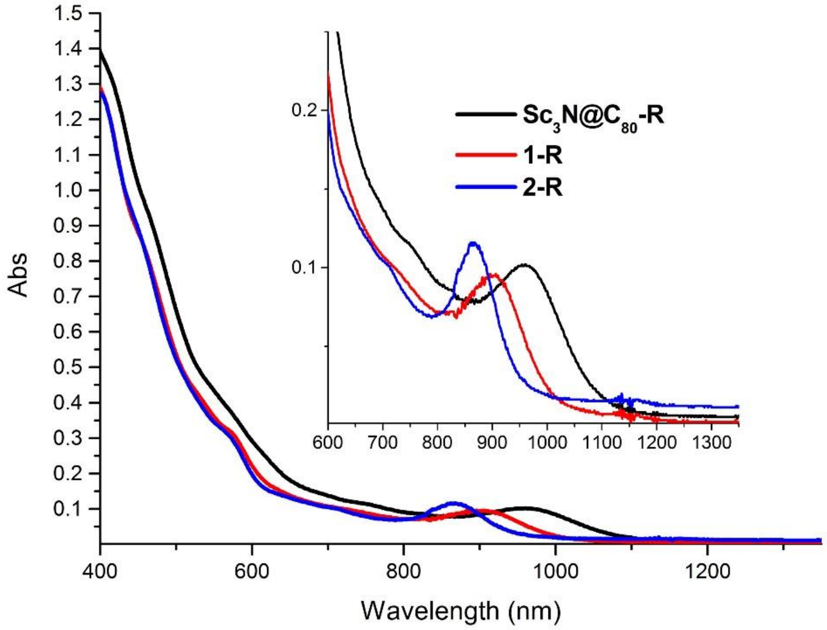

Figure S5. Vis-NIR absorption spectra of $Sc_3N@C_{80}$-R, **1-R**, and **2-R** in toluene solution.

Magnetic properties

Magnetic properties were measured using a Quantum Design VSM MPMS3 magnetometer. The sample drop-casted from toluene solution into a standard sample holder. To measure relaxation time in DC mode, the sample was first magnetized to the saturation at 3 T, then the field was swept as fast as possible to B = 0 T or B = 0.2 T, and then the decay of magnetization was recorded. Decay curves were fitted using stretched exponential function:

$$Y_{stretched} = A_0 e^{-\left(\frac{t}{\tau}\right)^{\beta}} + y_0$$

where τ is the relaxation time and y_0 is an equilibrium magnetization at the given field and temperature. See Ref. [1] for a detailed discussion of the determination of relaxation times by DC magnetometry

Table S1. Relaxation time of magnetization, τ_M, of **1-R**

T, K	τ_{M}, s	St. dev., s	β
1.8	81039.2	1525.2	0.58
2.0	32393.0	1047.6	0.65
2.4	10054.5	50.9	0.64
3.0	1599.2	17.0	0.71
4.0	341.4	1.2	0.69
5.0	105.5	1.2	0.60

Table S2. Relaxation time of magnetization, τ_M, of **1**

T, K	τ_{M}, s	St. dev., s	β
2	70838.8	1089.4	0.55
2.2	20523.5	890.6	0.61
2.5	7588.8	75.6	0.66
3	1461.0	9.1	0.74
3.8	342.1	0.5	0.74
5	59.5	0.3	0.75

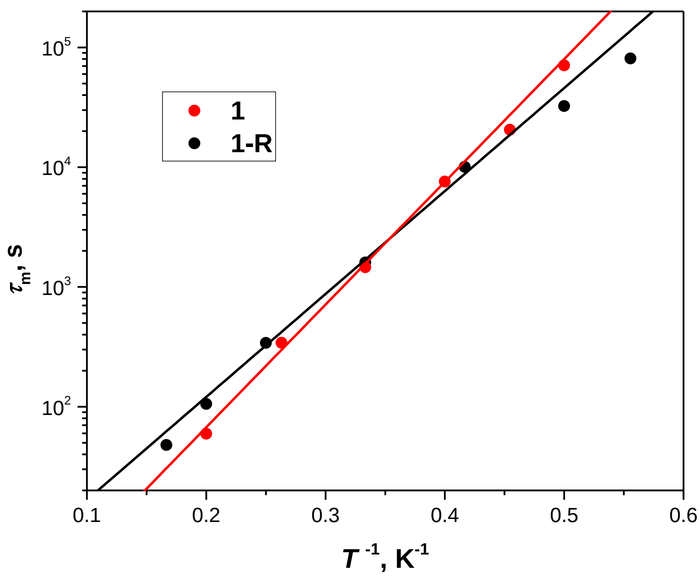

Figure S6. Relaxation time of **1-R** (black) and **1** (red) as a function of temperature, dots are experimental values, lines are fits with Orbach relaxation mechanism

Temperature dependence of the relaxation times of $DySc_2N@C_{80}$ (**1**) in the studied temperature range is virtually linear in Arrhenius coordinates, which points to the Orbach relaxation mechanism:

$$\tau_m^{-1}(T) = \tau_0^{-1}\exp(-U^{\mathrm{eff}}/T)$$

where U^{eff} is the effective barrier and is the attempt time. A good fit to the experimental data for **1** is obtained by an Orbach process with the U^{eff} of 23.6 ± 1 K and τ_0 of 0.6±0.2 s (see Ref. 1 for more details on the relaxation of magnetization in $DySc_2N@C_{80}$). For **1-R**, analogous fit gives the U^{eff} value of 19.8 ± 0.6 K and τ_0 of 2.3±0.4 s. Thus, 1-R has somewhat smaller barrier but longer attempt time, which leads to a slower decrease of the relaxation time with temperature and hence higher blocking temperature in the derivative. Both the sizes of the barrier and the prefactors are rather unusual. According to *ab initio* calculations, the Dy ion in $DySc_2N@C_{80}$ has a strong magnetic anisotropy and large crystal field splitting exceeding 1940 K. The first excited crystal-field state is predicted to be near 570 K. Thus, if the in-field relaxation of **1** and **1-R** at 2–5 K follows the Orbach mechanism, it cannot involve excited spin states. We already observed similar Orbach processes with low-energy barriers and long attempt times in some other fullerenes ($Dy_2S@C_{82}$,[2] $Dy_2@C_{80}$-CH_2Ph[3]) and hypothesized that they may correspond to the relaxation via low-frequency vibrations of the molecules. The fact that the Raman relaxation process with the local phonon mode may be observed as an Orbach process with the barrier corresponding to the phonon frequency has been realized back in 1960s.[4-6] A recent computational study of

the role of phonons in spin relaxation in SMMs showed that an anharmonic phonon with finite linewidth may lead to Orbach-like behavior with the effective barrier corresponding to one half of the phonon frequency.[7]

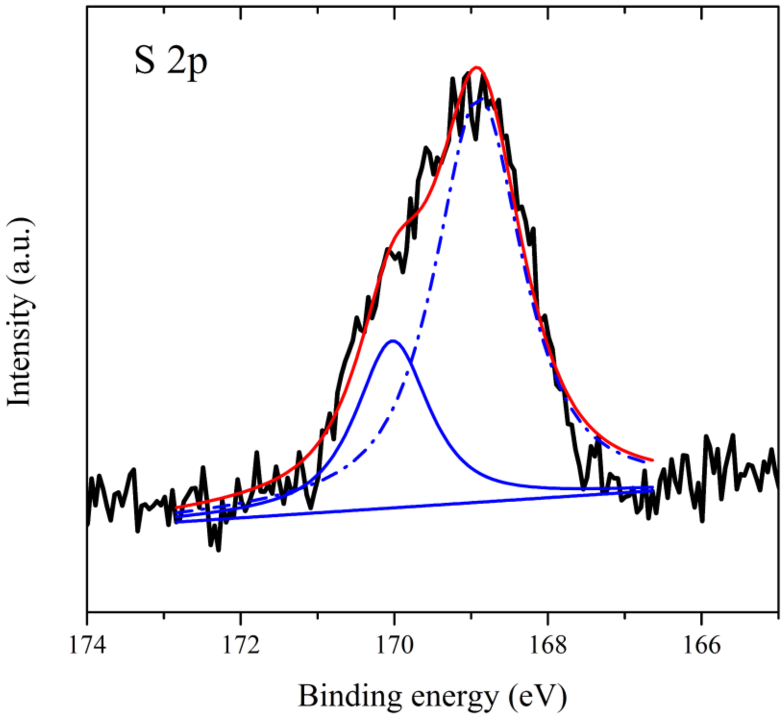

Figure S7. XPS spectrum of the **C_{60}-R** (analog of the EMF-based derivatives synthesized with C_{60}) on Au substrate in the S 2p energy range.

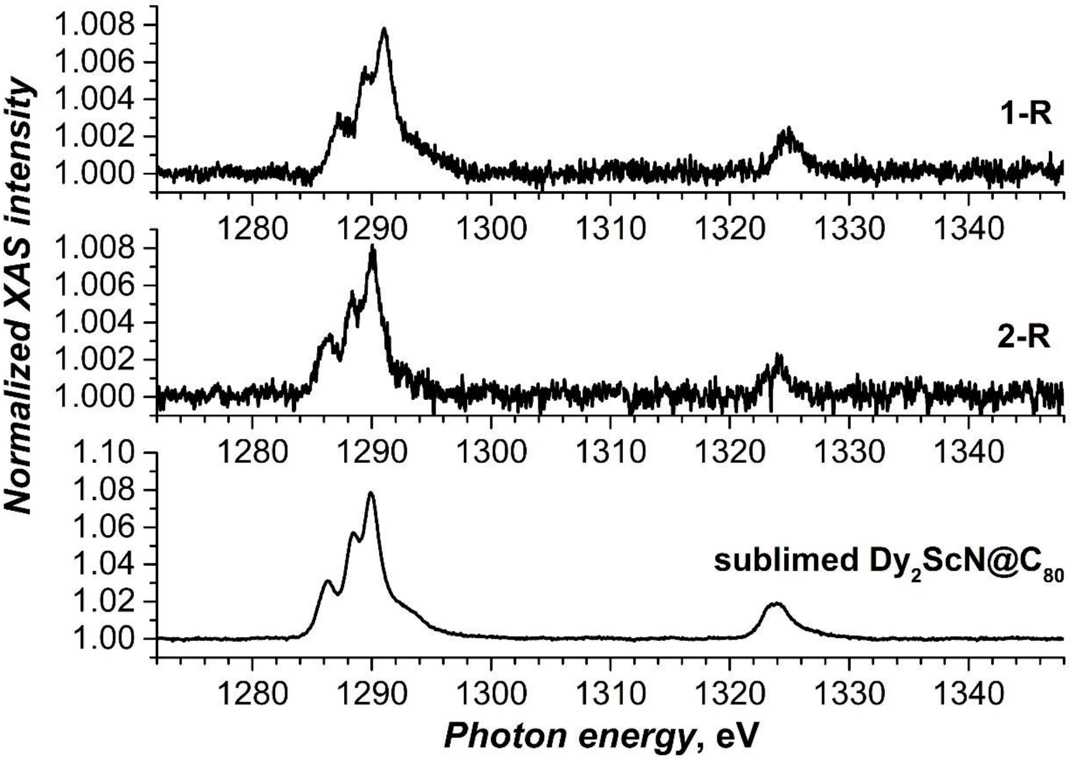

Figure S8. X-ray absorption spectra (XAS) of deposited **1-R** and **2-R** submonolayers as compared to the sublimed submonolayer of $Dy_2ScN@C_{80}$ (coverage of ca 50% as estimated by STM). XAS intensity is normalized to the signal at 1270 eV (i.e. out of the range of the Dy absorption). Based on the XAS intensity, we can conclude that the coverage of the **2-R** submonolayer is 10 times lower than in the sublimed sample and corresponds to ca 5% of the $Dy_2ScN@C_{80}$ monolayer (but note that derivatized molecules occupy larger area on the surface). XAS of **1-R** has similar intensity as **2-R**, but with only one Dy ion per molecule (versus two Dy ions in **2-R**), the coverage is 2 time higher, ca 10%.

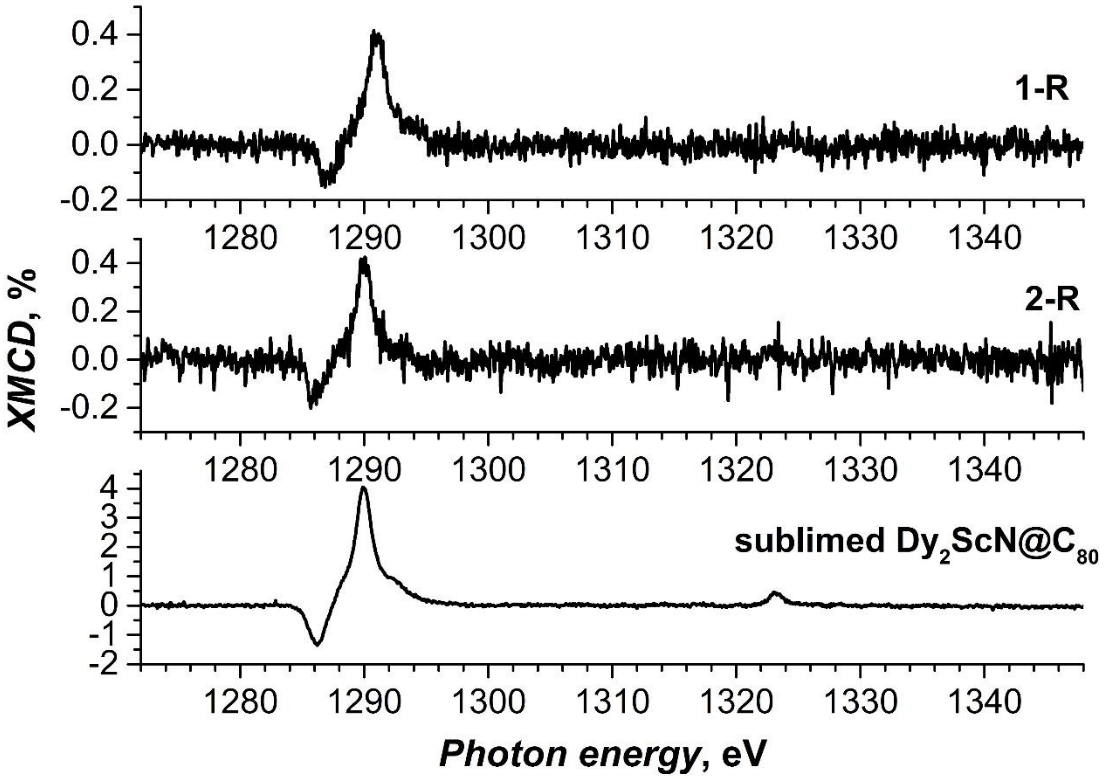

Figure S9. XMCD spectra of deposited **1-R** and **2-R** submonolayers as compared to the sublimed submonolayer of $Dy_2ScN@C_{80}$. Relative XMCD intensity is given in % and is computed as 100%*(I^+ − I^-)/(I^+ + I^-) (i.e. as assymetry), where I^+ and I^- are XAS intensities for circular polarized X-rays.

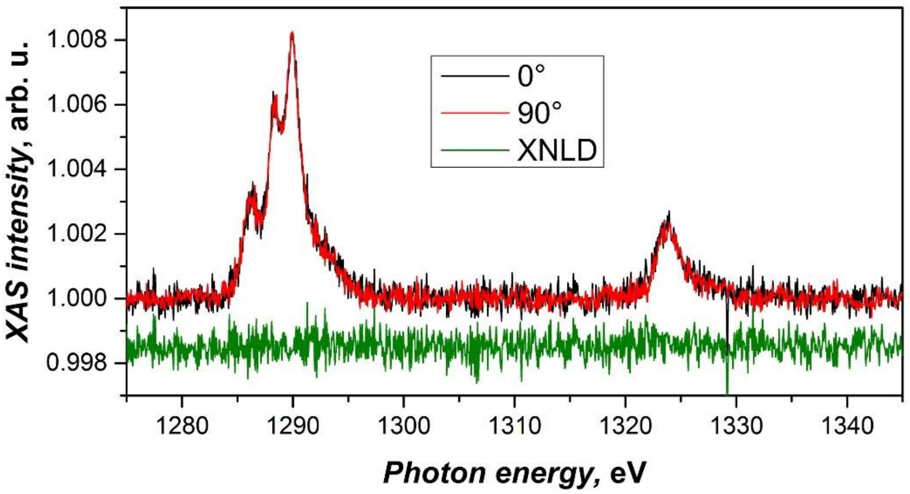

Figure S10. X-ray absorption spectra of the SAM of **2-R** on Au(111) measured with linear-polarized X-rays (black, red) and X-ray natural linear dichroism (green).

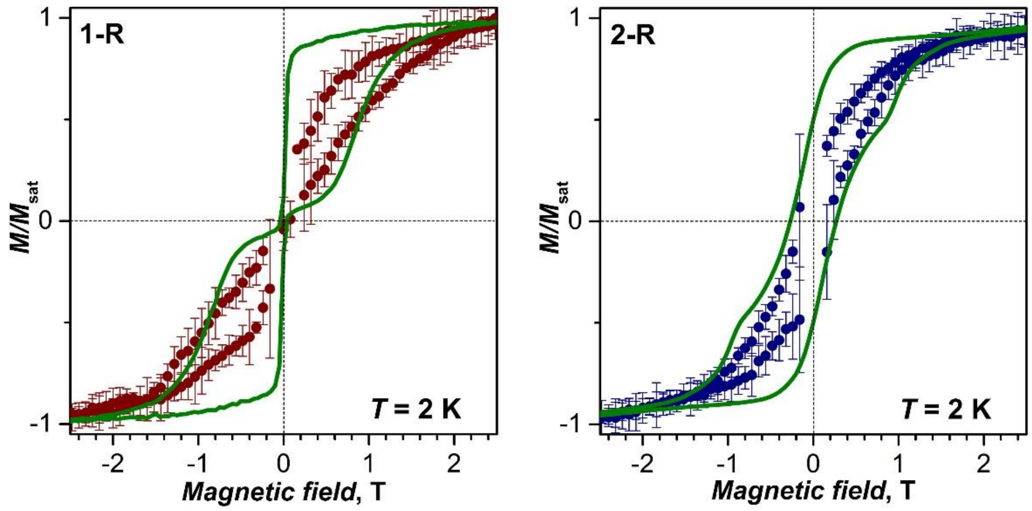

Figure S11. Magnetization curves of **1-R** (left) and 2-R (right) measured at 2 K by XMCD for submonolayers (dots with error bars, sweep rate 33 mT/s) and by SQUID magnetometry for powder samples (lines, sweep rate 2.9 mT/s). Note that the sweep rate in XMCD measurement is more than 10 time faster than in SQUID measurement. However, the magnetic hysteresis in submonolayer sample is still considerably narrower showing that the relaxation of magnetization is much faster for the molecules in the contact with Au.

Molecular Dynamics Modelling

Born–Oppenheimer molecular dynamics simulation were performed at the PBE[8]-D[9]/DZVP level theory (mesh cutoff 250 Ry) using CP2K code.[10-12] The velocity Verlet algorithm was employed with the time step of 0.5 fs and three Nosé–Hoover chain thermostat set at 300 K with the thermostat time constant 100 fs. The Au surface was fixed through the molecular dynamics modeling.

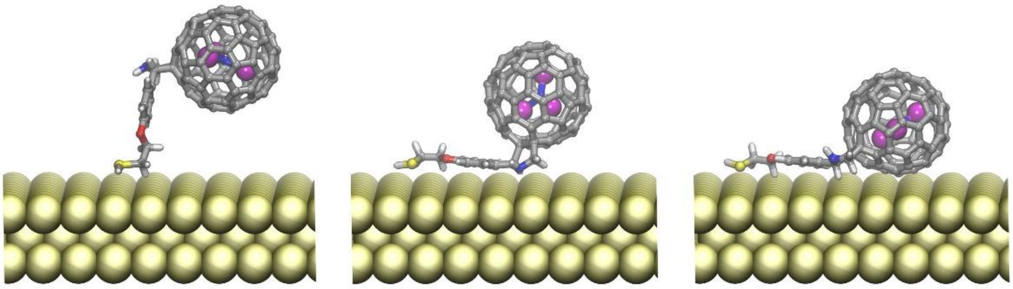

Fig. S12. Selected configurations of the $Sc_3N@C_{80}$-R' molecule along the MD trajectory: vertical structure (initial configuration), the structure after 2 ps (horizontal alignment of the linker and vertical position of the fullerene), and the structure after 5 ps (fully horizontal configuration)

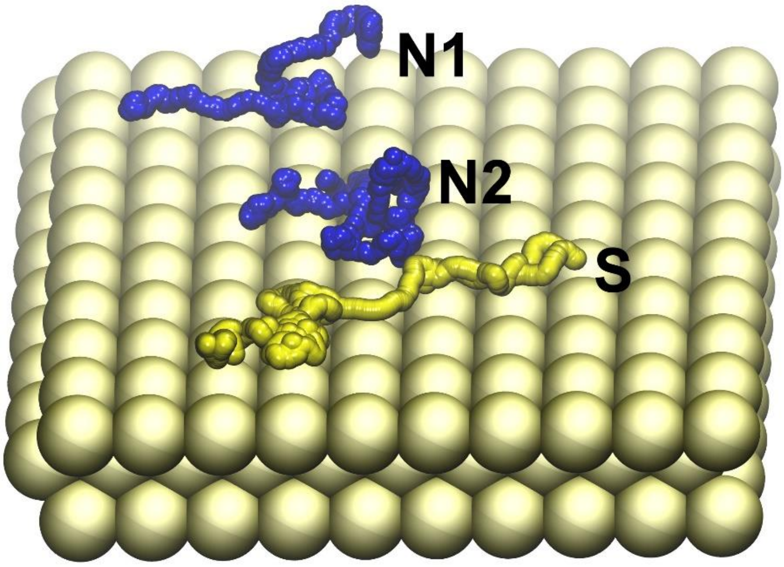

Figure S13. MD trajectory of $Sc_3N@C_{80}$-R' (20 ps after the molecule first acquired the fully horizontal configuration, 300 K, PBE-D/DZVP). Most of the atoms are omitted, shown are only sulfur and two nitrogens: N1 is the central atoms in the Sc_3N cluster, N2 is in the pyrrolidine ring.

DFT optimization of $Sc_3N@C_{80}$-R configurations on the Au surface

The structure optimization of different $Sc_3N@C_{80}$-R configurations on the Au surface at the PBE-D level was performed with the VASP code using projector augmented-wave (PAW) method.[13-15] Bader atomic charges were obtained by integrating the electron densities generated by VASP with the use of Bader code.[16, 17]

Table S3. Relative energies (total, ΔE_{tot}, and dispersion contribution, ΔE_{disp}) and changes of the Bader charges (ΔQ) of the $Sc_3N@C_{80}$-R molecule and its fragments after deposition on the Au surface in different configurations*

	ΔE_{tot}, eV	ΔE_{disp}, eV	$\Delta Q(Sc_3N)$	$\Delta Q(Sc_3N@C_{80})$	ΔQ(linker)	ΔQ(total)
1	2.94	3.27	-0.01	0.14	0.16	0.30
2	2.81	3.07	0.00	0.15	0.13	0.28
3	2.18	2.03	0.00	0.15	0.08	0.23
4	1.74	1.28	0.00	0.13	0.24	0.37
5	2.06	1.21	0.00	0.10	0.20	0.30
6	1.76	1.11	0.01	0.22	0.17	0.39
7	1.68	1.37	0.01	0.15	0.19	0.34
8	1.77	1.33	0.01	0.07	0.23	0.30
9	1.79	1.30	0.01	0.07	0.24	0.31
10	0.20	-0.31	-0.01	0.14	0.25	0.38
11	0.29	0.14	0.00	0.24	0.18	0.42
12	0.19	0.01	-0.02	0.32	0.15	0.47
13	0.21	0.24	0.00	0.24	0.16	0.40
14	0.07	0.01	-0.01	0.25	0.19	0.44
**15**	**0.00**	**0.00**	**-0.01**	**0.30**	**0.17**	**0.47**
16	0.27	0.35	0.00	0.20	0.22	0.42
17	0.16	-0.11	-0.01	0.14	0.27	0.41

*Structures 1 and 2 have vertical orientation of the $Sc_3N@C_{80}$-R molecule as in Fig. 4a, structures 3 – 9 have horizontal arrangement of the linker but vertical orientation of the fullerene core (i.e. no direct contact of $Sc_3N@C_{80}$ with the surface, as in Fig. 4b), whereas in structures 10 – 17 the fullerene is touching the metal surface (as in Fig. 4c)

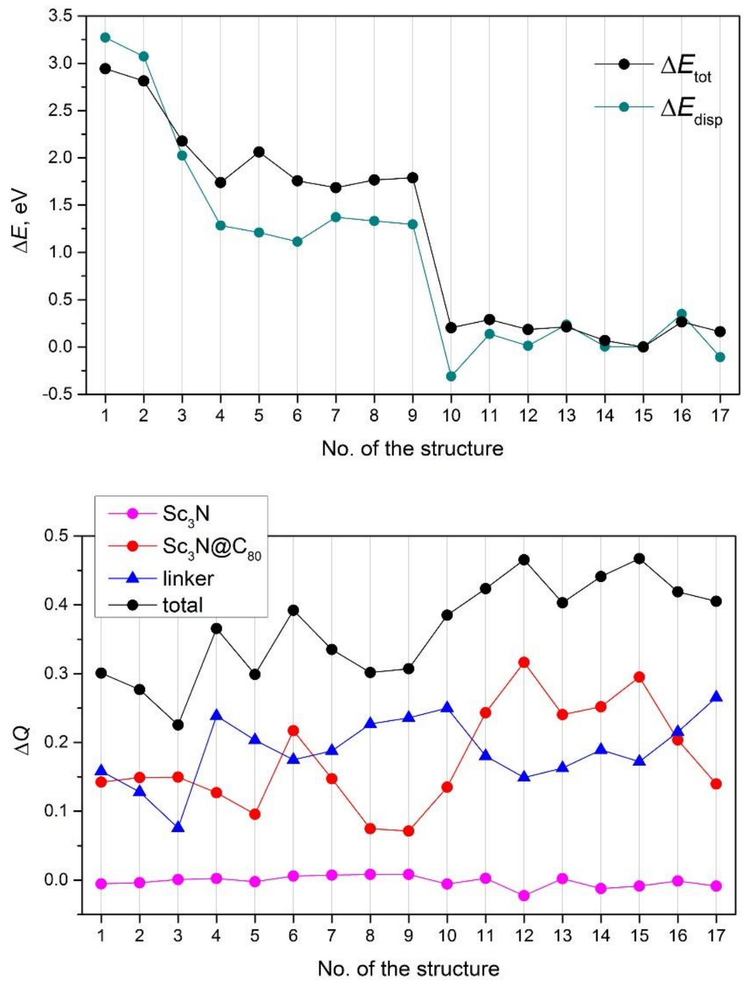

Figure S14. Top: relative energies of 17 optimized configurations of $Sc_3N@C_{80}$-R on Au. Bottom: changes of the Bader charges (with respect to the isolated $Sc_3N@C_{80}$-R molecule) after deposition on the Au surface

References

1. D. Krylov, F. Liu, A. Brandenburg, L. Spree, V. Bon, S. Kaskel, A. Wolter, B. Buchner, S. Avdoshenko and A. A. Popov, *Phys. Chem. Chem. Phys.*, 2018, **DOI: 10.1039/C8CP01608A**.
2. C.-H. Chen, D. S. Krylov, S. M. Avdoshenko, F. Liu, L. Spree, R. Yadav, A. Alvertis, L. Hozoi, K. Nenkov, A. Kostanyan, T. Greber, A. U. B. Wolter and A. A. Popov, *Chem. Sci.*, 2017, **8**, 6451-6465.
3. F. Liu, D. S. Krylov, L. Spree, S. M. Avdoshenko, N. A. Samoylova, M. Rosenkranz, A. Kostanyan, T. Greber, A. U. B. Wolter, B. Büchner and A. A. Popov, *Nat. Commun.*, 2017, **8**, 16098.
4. P. G. Klemens, *Phys. Rev.*, 1962, **125**, 1795-1798.
5. D. L. Mills, *Phys. Rev.*, 1966, **146**, 336-343.
6. M. B. Walker, *Phys. Rev.*, 1967, **162**, 199-208.
7. A. Lunghi, F. Totti, R. Sessoli and S. Sanvito, *Nat. Commun.*, 2017, **8**, 14620.
8. J. P. Perdew, K. Burke and M. Ernzerhof, *Phys. Rev. Lett.*, 1996, **77**, 3865-3868.
9. S. Grimme, J. Antony, S. Ehrlich and H. Krieg, *J. Chem. Phys.*, 2010, **132**, 154104.
10. J. Hutter, M. Iannuzzi, F. Schiffmann and J. VandeVondele, *WIREs Comput. Mol. Sci.*, 2014, **4**, 15-25.
11. J. VandeVondele and J. Hutter, *J. Chem. Phys.*, 2007, **127**, 114105.
12. J. VandeVondele, M. Krack, F. Mohamed, M. Parrinello, T. Chassaing and J. Hutter, *Comput. Phys. Commun.*, 2005, **167**, 103-128.
13. J. Hafner, *J. Comput. Chem.*, 2008, **29**, 2044-2078.
14. G. Kresse and J. Furthmüller, *Phys. Rev. B*, 1996, **54**, 11169-11186.
15. G. Kresse and D. Joubert, *Phys. Rev. B*, 1999, **59**, 1758-1775.
16. E. Sanville, S. D. Kenny, R. Smith and G. Henkelman, *J. Comput. Chem.*, 2007, **28**, 899-908.
17. G. Henkelman, A. Arnaldsson and H. Jónsson, *Comput. Mater. Sci.*, 2006, **36**, 354-360.
